# The impact of a set of measures on the incidence of central line-associated bloodsteam infection in intensive care units

**DOI:** 10.1186/1753-6561-5-S6-P57

**Published:** 2011-06-29

**Authors:** DP Cais, F Minenelli, ML Biancalana

**Affiliations:** 1 Infection Control, Hospital Samaritano, São Paulo, Brazil

## Introduction / objectives

Intravascular catheters are necessary in health care practice, particularly in Intensive Care Units (ICU). However, central line-associated bloodstream infection (CLABSI) is related to prolonged hospitalization, increase of mortality and costs.

### Aim

To analyze the impact of the implementation of a set of measures on the incidence of CLABSI.

## Methods

The study was conducted during 2010 in a general ICU and in a Cardiology ICU of a medium sized hospital in Sao Paulo, Brazil. CLABSI was defined according to the National Healthcare Safety Network definition. The set of measures was introduced in July 2010 and included reinforcement of prevention measures, switching opaque valve connectors to a transparent version, introduction of chlorhexidine gluconate transparent dressing, and weekly audits with feedback to the healthcare team on CLABSI prevention measures.

## Results

Analysis between January and July indicated development of infection related to the catheter’s maintenance and not to its placement, which directed the set of measures. Incidences of CLABSI before and after measures implementation were 5.1 and 3.8 per 1,000 catheters-day. The highest value was observed in July, 8.7, and a reduction occurred over the following 3 months: 4.0, 3.3 and 2.0. In November, an increase was noted (6.5) and observational audits of hand hygiene adherence were performed, leading to a reduction to 3.0 in December.

## Conclusion

The measures implementation contributed on the decrease of the incidence of CLABSI. However, to maintain low rates, continuous and multidisciplinary strategies need to be implemented focusing on motivation, education, monitoring, and dissemination of information. Furthermore, responsibilities need to be shared with local leaders.

## Disclosure of interest

None declared.

